# A Clinical Preventive Strategy Based on a Digital Tool to Improve Access to Endocrine Disruptors Exposure Prevention: The MEDPREVED Study

**DOI:** 10.3390/ijerph191911993

**Published:** 2022-09-22

**Authors:** Marion Albouy, Maud Parthenay, Maeva Nogues, Agathe Leyris, Léa Degorce, Zacharie Barthelemy, Diana Rafidison, Anne-Sophie Gourgues, Virginie Migeot, Jean Pylouster, Antoine Dupuis

**Affiliations:** 1 Faculty of Medicine and Pharmacy, University of Poitiers, 6 Rue de la Milétrie, 86000 Poitiers, France; 2 Ecology and Biology of Interaction, CNRS UMR 7267, CEDEX, 86073 Poitiers, France; 3 INSERM-CIC1402, University Hospital of Poitiers, 2 Rue de la Milétrie, CEDEX, 86021 Poitiers, France; 4 BioSPharm Pole, University Hospital of Poitiers, 2 Rue de la Milétrie, CEDEX, 86021 Poitiers, France; 5 Research Centre on Cognition and Learning, MSHS, 5 Rue T. Lefebvre, CEDEX, 86073 Poitiers, France

**Keywords:** endocrine disruptor, clinical prevention, general practitioners, midwives, paediatricians, health professionals, health education, environmental health

## Abstract

**Introduction**: The digitalized PREVED (PREgnancy, PreVention, Endocrine Disruptor) questionnaire was used in the clinical practices of health professionals (HP) who adhered to the MEDPREVED strategy. The objectives were to assess the strategy and to determine if it could improve access to endocrine disruptor (ED) exposure prevention. **Methods**: After having filled in the digital questionnaire in HP waiting rooms, patients were invited to talk about ED exposure during the consultation. HPs were previously trained in ED and had received a prevention kit for their patients. After the seven-month implementation phase, the evaluation phase consisted of five mixed assessments: interviews with: (i) patients who were young children’s parents; (ii) patients in the general population; (iii) paediatricians; (iv) midwives; and a quantitative study on GPs. Assessment concerned feasibility, accessibility, and usefulness of the strategy; we then used the Levesque model to evaluate how it could improve access to ED exposure prevention. **Results**: The study included 69 participants. The strategy appeared feasible for the filling-out step due to digital and environment access. However, it depended on patient and HP profiles. The strategy seemed useful insofar as it facilitated reflexive investment, an intention to healthy behaviour and, rather rarely, talk about ED exposure. The beginning of this discussion depended on time, prioritizing of the topic and HP profile. The strategy has confirmed the Levesque model’s limiting factors and levers to access ED prevention. **Conclusions**: The MEDPREVED strategy is feasible, accessible, and useful in clinical prevention practice. Further study is needed to measure the impact on knowledge, risk perception and behavior of beneficiaries of the MEDPREVED strategy in the medium and long term.

## 1. Introduction

Endocrine disruptors (ED) are defined as “exogenous substances or mixtures that alter function (s) of the endocrine system and consequently causes adverse health effects in an intact organism, or its progeny, or (sub)populations”. They are widely distributed, and even at very low doses, EDs are likely to have endocrine-disrupting effects [1]. People are exposed to many Eds, such as bisphenol A (BPA) through plastics [2], or parabens (PB) through cosmetics and personal care products [3]. Exposure to ED during pregnancy is likely to have negative health consequences as described by the Developmental Origins Hypothesis of Health and Diseases (DOHAD). They affect fetal development (nervous system disturbance, prematurity) and the fetus’ future life (behavioral disturbances, early puberty) [1].

Early interventions to reduce ED exposure significantly reduce this risk [4]; preventing exposure to ED must be a priority for health professionals (HP) [5]. A few studies have examined how to limit ED exposure through restrictive diet [6,7,8] or perinatal health education program. One of the perinatal education programs, is called “PREVED” (PRegnancy prEVention Endocrine Disruptors) [9,10,11]. PREVED uses an ED exposure assessment tool which consists of 33 questions exploring self-esteem, perceived health, health care renunciation and risk aversion, risk perception, knowledge about ED (routes and sources of exposure, ability to name some ED molecules or families of molecules, and definition of an ED), ED risk perception (perceived severity and vulnerability), expectations of a healthy baby, trusted person, level of concern about risks related to pregnancy, relation to risk visibility and perceived ability to reduce one’s exposure to ED with a visual analogic scale. Height questions explore the knowledge, attitude, and practice (KAP) dimensions, which identify knowledge level, attitudes linked to behavior change, preventive practices. Indeed, as it is often difficult to link knowledge to healthy behaviour, it is important to take into account the three psychological components of patient behavior (cognitive, emotional and conative) [12].

Among actors who could educate pregnant women, health professionals (HP) have had greater medical endorsement than non-medical professionals [11] and clinical prevention practices are integrated into medical practice [13]. In these prevention practices, HPs can use educational tools (e.g., pamphlets, interviews, speaking groups, and workshops), methods highlighting active listening [11] and questionnaires on different topics [14,15,16,17,18].

Some of these questionnaires use digital technology, or mhealth, such as Internet websites [19,20] accessible on smartphone. Computer software and digital questionnaires help to identify health risk behaviours, such as misuse of alcohol, smoking, lack of physical activity, after which, the doctor comments to the patient about the results, gives necessary information about the risks and tries to change the patient’s behaviour [21]. However, these tools adopt a prevention approach to risk reduction that does not always empower the patient, so other tools, filled in by the patient, are used in an asset-based approach. Indeed, paediatricians, midwives and GPs intentionally use their waiting rooms to disseminate a broad range of health-related information, sometimes with mhealth. However, some authors have shown that often there are no clearly defined prevention strategies in this workplace [22]. 

To our knowledge, there exists no study assessing digital tool on ED exposure prevention. Following the hypothesis that patient should be conscious of their life habits and their health impacts of them, and that new knowledge conveyed by HPs could modify attitudes and behaviour, we have developed a strategy for ED exposure prevention based on a simplified digital tool from the PREVED questionnaire, available in waiting room and integrated into clinical preventive practices. We have implemented the strategy named “MEDPREVED” and evaluated it from the standpoints of accessibility, feasibility, and usefulness, after which we have tried to determine whether the new strategy could improve access to ED exposure prevention.

### Methods

The strategy was based on the patient empowerment and motivational approach with self-assessment of life habits, especially regarding ED and a brief intervention supported by HP training on ED and HP equipment with a prevention kit. Empowerment is a process that enables people to make decisions and exercise control over their lives by developing a positive representation of themselves [23]. Brief intervention is similar to a reduced version of motivational maintenance, and can naturally find its place in general or paediatric medicine, where long-term monitoring and prevention are at the heart of their practices. It consists, often over several consultations, of a risk assessment of the subject, an exchange on the motivations for change and active participation, through open, non-judgmental questions. The patient plays a central role in his/her own decision to change. It is a responsible rather than a paternalistic approach. 

For the **development phase** we carried out a pilot study on GPs, which allowed us to adapt the strategy because there were problems recruiting patients and the questionnaire was perceived as too complex to be filled out by patients. As a result, the PREVED questionnaire was simplified in order to respect the health literacy concept in accordance with the “Facile À Lire et à Comprendre” criteria [24]. It included only eight brief questions on: (i) the participant’s ED knowledge about routes and sources of exposure, ED definition, and family names; (ii) perception of risk, especially perceived severity; (iii) patient practices (behaviours) through ED eviction and ways of achieving it. Finally, to induce patient recruitment, the questionnaire was digitalized with a QR code on posters in the waiting rooms of the different HPs. 

For the **implementation phase**, HP recruitment was carried out through HP councils, the infant and maternal protection units of departmental council of five French departments, and the University Masters local associations of the of Nouvelle-Aquitaine region. The HP regional college (“Union Régionale des Professionnels de Santé” or URPS) in Nouvelle-Aquitaine and a pesticide control association of GPs have also agreed to spread the message of the study to GPs with major paediatric activity, as well as private and hospital paediatricians. Approximately 100 HPs were contacted by the researchers by email, telephone or directly at the place of practice. Moreover, midwives were contacted through midwives of the Nouvelle Aquitaine professional order.

Those who were interested in participating in the study were invited to send an email expressing their interest. A reply email was sent to them containing the entire MEDPREVED strategy (kits and display) along with the protocol. Recruitment of HPs began on 13 April 2021 and ended on 12 November 2021. The implementation phase began on 13 April 2021and ended on 31 December 2021.

After having filled in the digital questionnaire on their smartphone in waiting rooms, patients were invited to talk about ED exposure during their consultation with their HP. While we did not collect the patients’ answers to the PREVED questionnaire, we made their responses available to the HP during the consultation if the patient so wished, in order to initiate a discussion on ED exposure prevention. HP were previously trained on ED with a training kit and had received a prevention kit with brochures for their patients. The self-training kit included: a « 2 min all inclusive» video made by the eSET (Health Environment for All) of Bourgogne Franche-Comté [25], a link to the INSERM information package on ED [26], an article by the International Federation of Gynecology and Obstetrics (FIGO) [5], and a user’s guide for private physicians, produced by the URPS of Provence-Alpes-Côte d’Azur (PACA) [27]. The prevention kit given to the patient consisted of: the PACA URPS Guide, the same video, a guide by the Agence Régionale de Santé (ARS) Nouvelle-Aquitaine [28], a flyer from ARS Nouvelle Aquitaine [29], an application “Ma Maison Santé” created by the French Mutualité Pays de la Loire [30] and a link to the WECF (Women Engage for a Common Future) France website [31].

After the seven month implementation phase, the **evaluation phase** was composed of five assessments: four using a qualitative approach with interviews of: (i) patients who were young children’s parents; (ii) patients in the general population; (iii) paediatricians; (iv) midwives; and one with a quantitative approach of GPs. The patients were recruited on a voluntary basis by asking them to fill in their email at the end of the questionnaire: if they accepted, they were contacted again by two medical students (LD, MP) for a short telephone interview. The data were then anonymized. The analysis and coding of the qualitative data were done by triangulating content analysis. For the quantitative part, self-assessment was hetero-administered via Limesurvey©. (Version 4.3. Hambourg, Germany)Statistical analysis of the data was carried out without software. Qualitative variables were described in terms of numbers and percentages and quantitative variables in terms of means, standard deviation, minimum and maximum.

Using the Levesque model, we then tried to determine whether the MEDPREVED strategy could improve access to ED exposure prevention. This model presents the determinants related to the patient and the determinants related to the health system, which are of interest throughout the patient’s care pathway and may or may not result in access to health services. It is determined not only by the patient’s ability to perceive their health needs, to seek, reach, pay for and engage in health activities, but also by the characteristics of the health system: ease of access, acceptability and the availability of the service, affordability and relevance [32].

## 2. Results

### 2.1. Study Population

The study population included 69 participants. Among the hundreds of HPs contacted, eight paediatricians or GPs with a paediatric activity (Pe1 to Pe12), 26 GPs and 12 midwifes (M1 to M12) agreed to participate in the evaluation phase while of the 23 patients filled in the questionnaire, 11 included general population group patients (P1 to P11) and 12 young children’s parents (Pa1 to Pa12) agreed to participate to the evaluation phase. Characteristics of the 43 participants (23 patients, 12 midwifes and eight paediatricians or GPs with a paediatric activity) are described in Table 1. Characteristics of the 26 GPs are described in Table 2. The mean age of GP was 45 ± 13 (from 30 to 71) years. The mean number of medical practices’ duration was 16 ± 14 (from 1 to 48) years. Their responses in terms of feasibility, accessibility and utility of MEDPREVED strategy are presented in Table 3.

### 2.2. Feasibility and Accessibility of MEDPREVED Strategy

**Figure 1:**
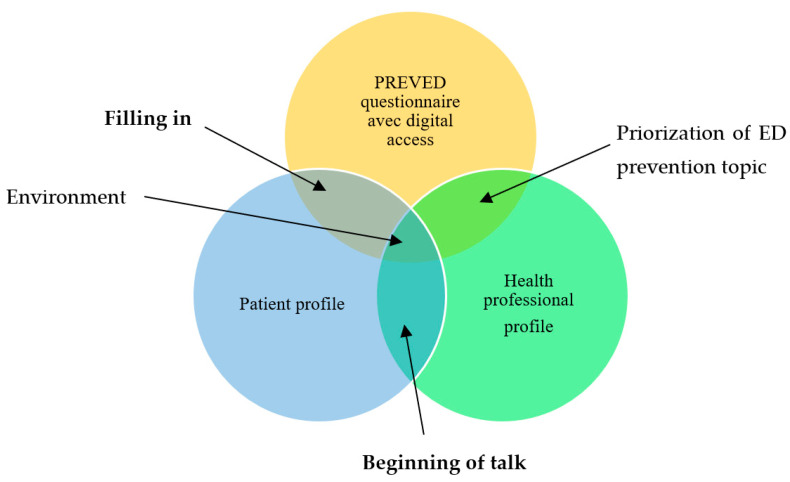
Modalities of MEDPREVED feasibility and accessibility.

We have summarized the modalities of MEDPREVED feasibility and accessibility in Figure 1. The interface between the digital tool and the patient profile refers to the filling-in of the questionnaire. The interface between the digital tool and the HP’s profile refers to the professional’s prioritizing of ED prevention topics in his/her practice. The interface between patient and HP profiles refers to the beginning of the discussion. Results of each modality are presented below.

In the MEDPREVED strategy, we judged feasible the filling in of the questionnaire, but not the beginning of the talk about ED exposure prevention, notably because of non-prioritizing of ED prevention among HPs.

1. Feasibility of the filling in was permitted by digital accessibility, PREVED questionnaire accessibility, patient and physician profiles and the environment.
  (a) Digital accessibility, through digital technology with a QR code, facilitated complete of the questionnaire, at times in different time-steps, via smartphone use universality, COVID-19 context facilitator and digital ease of use.
    - Smartphone use universality: *“I think they all have a smartphone”* (Pe3); *“They all have the phone that goes with it to be able to do the right thing”* (M3)
    - Covid19 facilitator: *“Given the context in which we endlessly scan QR codes: very accessible”* (Pa7).
    - Digital literacy and ease of use: *“I found that with the QR code system it’s really not bad”* (P3); *“I found it simple, fast”* (Pa8)
    - Digital preference: *“I would have put the paper in my bag, I would not have done it right away in the waiting room, I don’t think I would have done it”* (P6); *“I thought the QR code was pretty good and it avoided having to fill out a piece of paper it was easier to do because there was just to scan and then there was the questionnaire to display and then click on send and it was good.”* (M8)

However, some participants noted the side effects of technology: unequal access due to age, culture, lack of internet access and paper preference:

    - Age: *“If my mother had to fill it out, she wouldn’t have a suitable phone. 'Fine, that’s fine... it’s more complicated for some people”* (Pa1)
    - Culture: *“People don’t yet have QR code education with us”* (Pe3).
    - Non-internet access: *“I don’t always have internet”* (P7); *“As soon as it is digital I find that it is... a bit excluding because there are always people who don’t have internet on their phone”* (Pa6)
    - Paper preference: *“I find that understanding on a piece of paper is much easier and makes me want to do it a lot more than picking up the phone”* (P10); *“It’s not my area of choice the QR code”* (M12)

  (b) Feasibility was helped by the accessibility of the PREVED questionnaire with it associated its form and content. Mainly, feasibility seemed to depend on patient and physician profiles.

The form characteristics of the questionnaire were its simpleness, quickness and flow:

    - Simple: *“It’s super simple even.”* (M2); *“I know I have patients who told me it was simple to do, yes and not long”* (M8)
    - Quick: *“A questionnaire that is quick, easy to do”* (Pa11)
    - Flowing: *“Frankly it was fluid”* (Pa5); *“I liked it was a bit playful euh the fact of... sliding to put in order there, for the rooms of the house...”* (Pa10)

The contents of the questionnaire addressed health literacy with concrete but maybe too general questions, lacking in precision:

    - Questionnaire Literacy: *“I found it simple to understand and uh... no it was fine”* (Pa6);
    - Only one patient found it difficult: *“too medical lexicon”* (P10)
    - Concrete questions: *“I recognize myself with some plastic utensils, uh... with uh... here with product choices so it’s... oh yes we... right away we recognize our daily life»* (P7).
    - Lack of precision: *“I found it rather generalist, I mean not ultra-precise”* (Pa2); *“I find it unfortunate that at the beginning of the questionnaire it is not... I mean that the definition is not given to us before asking us questions about what we think about it”* (P10).

Mainly, feasibility seemed to depend on patient and physician profiles.

Patient profiles were described in terms of being informed, having previous hormonal disease, profession, parenthood, rurality, psychosocial characteristics (fatalism, risk perception, fear of judgment), interest and curiosity, ED exposure prevention behaviour:

    - Informed by social media: *“I think everyone has heard about it in the press at least”* (Pe7); *“it is true that we are hearing more and more about endocrine disruptors”* (P4)
    - Previous hormonal disease: *“I am particularly aware of this because I had hormone-dependent breast cancer”* (P1); *“maybe also because I have my daughter that I see she has early signs of puberty”* (P5); *“My father has myelodysplasia so I am even more aware today”* (Pa5); *“there are patients who are already informed, which is a limit that tells you more than you already know about the subject”* (M3)
    - Profession: *“My year as a child care worker involved a lot of prevention in this area”* (P2); *“I was very sensitized by my pharmacist colleague who works on bisphenol A”* (Pa12)
    - Rurality: *“I think that here in the countryside we still have a population that is, uh… interested in it anyway”* (P1)
    - Pregnancy-parenthood: *“my midwife told me about it and then I was told about it in the hospital, I think”* (Pa2)
    - Fatalism: *“at the same time a feeling of helplessness…there are still many things you can’t get your hands on”* (Pa4)
    - Risk perception: *“It can still be dangerous”* (Pa1); *“It’s hard, very hard to quantify... uh... that’s something that’s hard to actually touch, to palpate”* (P5)
    - Fear of judgment: *“When you left stuff with which you can write, I didn’t have much to say because I was afraid of saying something stupid”* (Pa5).
    - Interest and curiosity: *“I could talk for hours about this I think so much that I’m interested”* (P2); *“Those who told me about it were already fairly aware”* (Pe6); *“they still have a some curiosity”* (M11)
    - ED exposure prevention behavior: *“I had already changed a lot in terms of cosmetics since the beginning of my pregnancy”* (Pa4); *“It’s true that I have a tendency to... so I buy a little more organic than before”* (Pa5); *“I avoid everything aluminum, cellophane, things like that”* (P2).

Physician profiles were described in terms of clinical case experience, personal norms and stereotypes:

    - Clinical case experience: *“When we see in consultation boys with micro penises, […] or other pathologies where we can possibly suggest endocrine disrupters”* (Pe7); *“The parents I spoke to were either from a wealthy background or health professionals”* (Pe6).
    - Personal norms: *“I’m not too bobo bio bike quinoa. It is not my culture”* (Pe3); *“I would say that it could be useful but not to all audiences, we are the audiences who do not master the reading”* (M11)
    - Stereotypes: *“The parents here are, from an often very precarious environment and I think they don’t care”* (Pe2); *“It’s more of a middle-of-the-road thing a little bit, a little intellectual, those who ask themselves a lot of ecological questions”* (Pe5); *“We still have a diverse population, endocrine disruptors, it’s something of an elite problem”* (M6)

  (c) The environment of the MEDPREVED strategy was important: filling in at home or in the waiting room, what mattered was a quiet environment and the time needed.
    - Calm places: *“it was pretty quiet so I could concentrate on answering these questions”* (P2); *“it’s better [to do it in the waiting room] because once I went home I wouldn’t have had time to do it”* (Pa12); *“In the waiting room you have to manage the child, not sure you can complete a questionnaire at the same time”* (Pe6)
    - To have time: *“it was a way to wait, it was very good”* (Pa5); *“it keeps them busy in the meantime, it’s not stupid because they are all on their mobile phones in the waiting room”* (M2); *“I try not to be late so in the waiting room there is not too much waiting either”* (Pe7); *“It must also depend on the waiting rooms, at home they wait quite little, it is exceptional that they wait even 20minutes, I am not too late”* (M10).

The waiting room is an adapted place for prevention because posters are attractive, but not for all because of infobesity

    - Attractive: *“That’s exactly what attracted me, when I saw the QR code, in the waiting room I thought, Well, I’ll look where it leads”* (Pa12).
    - Infobesity: *“There are too many signs in waiting rooms, it won’t get enough attention for me”* (Pe2); *“Not sure people are taking the time to read the posters”* (Pe6)
    - Covid19: *“Heu…. It’s true that right now it’s not the best place because of COVID”* (M9)
    - Alternatives: *“I pasted the QR code on my desk”* (Pe7)

2. The beginning of talk about ED exposure prevention seemed not feasible, mainly because of lack of time and opportunity to prioritize the subject:
    - Lack of time: *“we don’t have much time with the doctor so we won’t take twenty minutes here to discuss”* (P5); *“not the time and not the money and a consultation of two hours paid 25 euros, it doesn’t interest me anymore”* (Pe1); *“Don’t want to talk about endocrine disrupters when I already know I’m going to be late”* (Pe5)
    - Lack of prioritizing: *“It doesn’t seem to me to be the priority of priorities (…) in 20 minutes I really deal with priorities of priorities”*(Pe5); *“Smoking, screens, food… I have lots of hobbies”* (Pe1); *“there are other things to talk about when you first see people”* (M11); *“I’m talking about endocrine disrupters like I’m talking about toxoplasmosis, lifestyle, sports and really it’s part of the three-quarter-hour consultation, that’s really it”* (M1).

Moreover, the profiles of physician such as his/her openness, lack of training or science scepticism influence feasibility:

    - Physician openness: *“I am quite aware as an individual, it is a subject that touches me a lot”* (Pe4)
    - Lack of training and legitimacy: *“the problem is also that we are not sufficiently trained”* (Pe2); *“I don’t feel like giving prevention speeches on a subject I don’t master”* (Pe2); *“Even during the studies we had one or two courses, I don’t even know by whom, nor in what subject”* (M8)
    - Skepticism: *“the lack of reliable studies and (…) is a bit like pediatrics and eating? with the impression that every two years there is a new trick that comes out, that contradicts itself a bit”* (Pe1); *“We are still in a kind of blur I find”* (Pe4)
    - Relationship: *“she played a somewhat more reassuring role”* (P3).

### 2.3. Usefulness

The principal uses of the MEDPREVED strategy are reflexive investment, the intention to maintain or adopt healthy behaviour and, the beginning of talk about ED exposure prevention.

- Personal reflexive investment: *“it made me think a lot at night”* (P1); *“there I really realize that I have no control over...”* (P11); *“I think that the questionnaire should arouse people’s curiosity and that’s good”* (Pe2); *“The idea of the questionnaire as it is a great idea, it can help to start the discussion”* (Pe2); *“I had forgotten but it allowed me to... get it in my head”* (Pa10); *“they found it interesting for the most part, many of them told me what! […] but it was for them, something they discovered”* (M9).
- Healthy behavior to maintain or to adopt: *“Maybe in the future, maybe pay more attention to what you buy”* (P8); *“It revives good habits”* (Pa12); *“...it made me do something I’ve been thinking about for a long time, which is buying a filter for drinking water”* (Pa4).
- Talk with his/her entourage: *“I realize when I talk about it, now I realize that everyone is in the same level of knowledge as me”* (P3).
- Beginning of talk about ED exposure with HP: *“it makes us go to the doctor because I think that if the doctor himself has to go to each person it can take him time so …”* (P8); *“it could be a very good “hook” for a talk”* (Pe1).
- Talk could help to change: *“The doctor asked me what I was washing her backside with […] I bought a... a supermarket thing you know the supermarket pchit-pchit there. She said, “Well, that’s not great”. There you go. So, it’s true that after I got home she said, “water’s better,” so I replaced that little thing with water (laughs)”* (Pa5).
- But it is rarely done: *“I didn’t think to talk to the doctor after”* (P5); *“Behind it you have to have answers to offer them what (…) If you don’t have solutions to propose behind…”* (Pe4); *I have no concrete feedback because I don’t necessarily talk about it* (M3).
- Collective reflexive Investment: *“We’re not super informed about uh...about all that”* (P6).

The questionnaire’s form and contents were criticized, and its anxiety-provoking aspect was highlighted:

- Form: *“Is it useful to answer a questionnaire when we won’t have the answers and information... yeah I don’t know?”* (Pa1); *“it is true that in the end I expected to have a little doc’ to just uh... we will say recall essential points and even if we can discuss it after...”* (Pa9); *“But then you don’t have the answer”* (M7).
- Content: *“because there were no answers, it leads to more questions than answers”* (P9); *“I feel like I’m still misinformed, actually”* (Pa4).
- Anxiety-provoking or guilt feeling or powerlessness: *“it’s hard to spend time eating well, eating healthy, eating organic… There is this time constraint”* (P3); *“Rather guilty, almost distressing, when you have to question everything you eat, buy”* (Pe6); *“One thing that keeps me from talking to parents is the anxiety side”* (Pe4).

Some solutions are highlighted: (i) the central role of all HPs, who must be trained about environmental health and health education methods, during dedicated consultations using the PREVED questionnaire for priority populations; (ii) the prevention kit, which has been particularly appreciated by patients; (iii) the prevention campaign.

- HP role priority: *“It would indeed reassure me to have very precise information coming from a medical authority”* (Pa4); *“if it is already something given by health professionals, we can really have confidence”* (Pa11).
- HP training on ED topic and on health education methods: *“I also think we need to adapt it…to the audience we have, because we can scare everyone after the person who is starving if we tell them that in addition to buying their canned goods, they are poisoning themselves … that’s not a good message either”* (Pa12).
- Dedicated consultations: *“We should really talk about it during the follow-up consultation of the “x” months or gender at the annual consultation”* (Pe2); *“Ah, it must not be at the same time as anything else, I think. It has to be dedicated to that”* (Pa6); *“Since I can’t organize that at every consultation, basically the dedicated consultation would be a good idea”* (M9).
- Or not: *“I think that [a fully dedicated consultation] could really stress me out.”* (Pa10); *“Birth preparation classes are about feeding the baby so there could be...a course on the baby’s environment because we’re talking in the classes about life after birth”* (Pa6); *“It’s complicated to do the whole thing on top”* (M1).
- By all HP: *“The pediatrician has a role to play”* (Pe6) *-“it should be done as soon as patients know they are pregnant (…) should intervene early”* (Pe1); *“Other professionals also have a say: in antenatal consultations midwives and gynecologists must brief parents well upstream, before we do”* (Pe6); *“pediatricians or doctors can play their role, especially in the patient-caregiver relationship”* (Pa7).
- Questionnaire systematically for one and all: *“give a brochure systematically with the QR code written on it”* (Pe2); *“adults are also affected by endocrine disrupters”* (Pa1); *“Maybe there should be flyers with QR code given in addition to consultation”* (M1).
- On the contrary, target a specific population: *“People who are really interested in it don’t need the doctor to tell them about it”* (Pe5); *“You need to talk about it more,... some people have heard the word but don’t necessarily know”* (Pa6).
- Consulting group: *“After no kind of Tupperware meeting and nobody will ever go”* (Pa5).
- Prevention kit: *“There is one that has images, which is much simpler, has much less text and is much more telling”* (P10); *“Platelets with just simple things that they can do, that’s really what I want to go on”* (Pe4); *“I think it’s good, they take it because I have more”* (M10).
- Prevention campaign for the general population: *“There are not enough prevention campaigns, I think.”* (Pe6); *“There are a lot of things, prevention, when it’s taught in school and it goes down the road and it works. But it means that it has to go through the State”* (Pa6).

Among HP, only five paediatricians had a patient who talked about ED exposure after having filled in the questionnaire versus 13 in GP.

Among training kit resources, GPs preferred the video (13/26), the URPS guide (8/26) and the Inserm website (2/26). Among prevention kit resources, GP preferred the ARS flyer (11/26), the video (10/26), the Mpedia guide (9/26) and the ARS guide (6/26). The regional website was preferred to the National one.

**Figure 2:**
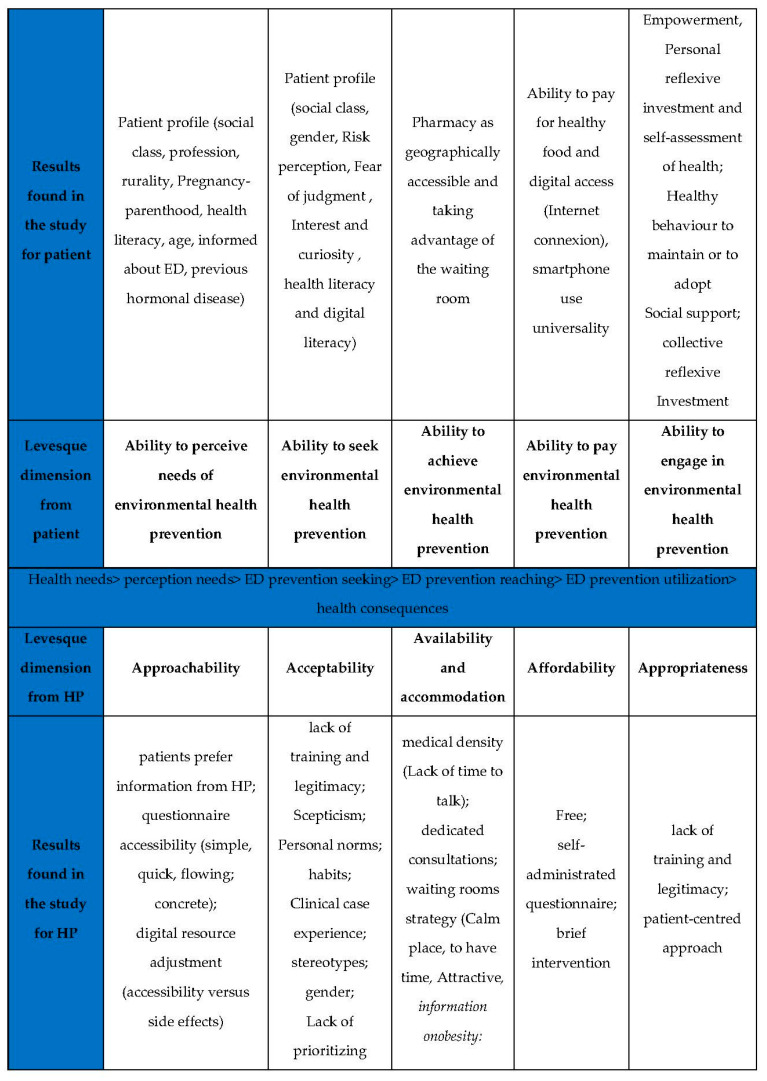
Results implemented in Levesque model.

From these results, we tried to use the Levesque model to highlight the determinants related to the patient and the determinants related to the health system in accessing the ED prevention (Figure 2). In the center of the figure, Levesque described health steps: health needs, perception of needs, ED prevention seeking, ED prevention reaching, ED prevention utilization and health consequences. For each step, he described dimensions from patient (upper part of figure) and dimension from HP (lower part of figure). We added the results found in the study for the patient (first line) and HP (last line) in front of these dimensions.

## 3. Discussion

In our study, we utilized Levesque’s patient dimensions (from 1 to 5) and professional dimensions (from 6 to 10) in examination of an environmental health prevention tool.

### 3.1. Ability to Perceive Needs of Environmental Health Prevention

From points of view expressed in this study, we found this to be a major determinant of MEDPREVED strategy use. Ability to perceive needs appeared associated with socio-professional level; the patient with lower status had less perception. Indeed, socio-professional level is known to be related to health literacy [33]: persons with low health literacy level are more vulnerable and exposed to environmental pollutants and less compliant to healthy lifestyles. Indeed, they are less inclined to use prevention or screening programs [34,35]. 

Moreover, younger persons seemed less inclined to perceive health environmental prevention needs then older persons. Younger persons, such as pregnant women or the parents of young children, are seen by health professionals as the target population to educate.

Finally, participants in our study were already well-informed about ED. Information and sensitization to health topics improves with social category, probably by better information dissemination in the upper social classes. Information is a determinant of risk perception on which behaviour change depends [36]. This result confirms that knowledge, beliefs, attitudes and practices of patients concerning this topic are a major determinant of ED prevention access.

### 3.2. Ability to Seek Environmental Health Prevention

From points of view expressed in this study, we found that the second Levesque patient dimension, the ability to seek ED prevention, is present and associated with patient profile such as social class, gender, health literacy and digital literacy.

In our study, patient participants were predominantly women, which is concordant with literature data showing that mothers are known to be more concerned than fathers about health [37].

In our study, while many patient participants predominantly found the PREVED questionnaire easy to understand, but some of them judged it overly complex, with too much medical jargon. This lack of health literacy could certainly affect the ability to seek environmental health prevention.

Moreover, our study used a digital tool to implement the PREVED questionnaire. This could have affected persons with low digital literacy. Indeed, persons with low literacy level have less access to digital tools and are less likely to use or perceive health information technology tools as easy or useful [38]. Illectronism, which increased with the COVID-19 crisis, is a key element of this Leveque dimension, as it defines the digital divide, which increases health inequalities. In France, in 2022, 30% of persons more than 65 years of age do not have Internet [39]. It is essential to locate these situations so as to avoid care renouncement.

### 3.3. Ability to Achieve Environmental Health Prevention

From the points of view expressed in this study, we found that this third Levesque’s patient dimension, the ability to seek ED prevention, is present insofar as geographic location of the practice as a site for education is not a problem because it is near people’s homes. Indeed, patients visit health professionals for different health reasons and take advantage of the waiting room. Moreover, our study was carried out in both rural and urban areas with the same result.

Patient transport is not a problem because PREVED can be filled in everywhere through the QR code. It permits universal access and personal usefulness.

### 3.4. Ability to Pay for Environmental Health Prevention

From the points of view expressed in this study, we found that the ability to pay for environmental health prevention was rarely mentioned. As income is associated with the ability to pay for healthy food [40], and some health professionals highlighted the difficulty of educating low-income patients. Moreover, while filling in the PREVED questionnaire is free of charge, it requires digital access (Internet connection and smartphone).

### 3.5. Ability to Engage in ED Prevention

From the points of view expressed in this study, we found an ability to engage in environmental heath prevention through the empowerment concept. Our study has shown that the PREVED questionnaire develops self-reflection and self-assessment of health. By this reflexive approach, many participants have become actors in their prevention by changing some of their practices, while others have become aware of a real lack of knowledge and have deepened their knowledge of the subject using computer resources. 

One condition of this empowerment appears to be social support. Indeed, educating the whole family instead of one person is more efficient. Social support allows the patient to identify peers who can help them in a practical way in their behaviour change [41]. According to the health determinants model [42], social relationships and patient networks are among the most important factors with positive or negative influences on health. 

### 3.6. Approachability

Approachability refers to transparency outreach, information, screening. From the points of view expressed in this study, we identified a lever of approachability: patients preferred information from HPs or the government than other sources because they appeared more reliable than industrial sources [43,44].

Moreover, we identified a limitation factor regarding approachability: according to one person, the questionnaire was judged to be overly complex, with too much medical jargon. The PREVED questionnaire should perhaps be improved according to health literacy levels [33]. 

Approachability depends on digital resource adjustment (QR code). 

Patient are facing digital information through the Internet, which is a key source of accessible health information [45]. The Internet appeared in this study to have several advantages: it is convenient, democratized, universal, remotely accessible, usable, and fast to use, allowing users to pause when and where they want. This could be explained by a young survey population, corresponding to a generation accustomed to the digital [46]. Nowadays, digital applications are common in clinical prevention practices, for example for gestational age calculation [47], physical exercise measurement [48], cardiovascular risk factor measurement [49], weight loss measurement [50], skin cancer prevention [51] with an application where a person could be shown on a screen with the consequences of unprotected sun exposure on his or her face [52]. The smartphone can be useful in health promotion by encouraging a healthier lifestyle [53]. Patients who are satisfied with the digital form would be more likely to report health information about themselves on a regular basis, which could help in better monitoring and adaptation of advice from HP [54]. Moreover, in the pandemic context, the digital format appeared in the waiting room to be more hygienic compared to the paper format, which could be a vector of potentially pathogenic microorganisms [55].

However, several side effects have been demonstrated. Tools are not adapted for every age. For example, digital aids to discuss sexual health with HPs are acceptable and feasible to implement in younger populations but need testing with older patients so as to overcome uncomfortable feelings [56]. Tools are not adapted to low-income populations [57], and may even exacerbate health inequalities [58,59]. Moreover, they are sometimes anxiety-provoking [52]. While different supports, such as video-based digital health interventions (video clips), interactive tailored messages, graphics, are now possible, digital health education should follow the health education guidelines [60], with positive messages and video intervention in clinical settings [61]. For example, parents are five times less attentive to their child’s request for attention when using a telephone [62] and its use in a waiting room is a counter-productive message of reasoned smartphone utilization.

### 3.7. Acceptability

Acceptability refers to professionals’ values, norms, culture, and gender. From the points of view expressed in this study, we identified that HPs may have little belief in their effectiveness in prevention, even though prevention requires belief in its effectiveness [63], unfortunately, training in health education and in environmental health are limited during curricula. Indeed, several studies of American paediatricians showed that while they were interested to include environmental health in their practices, they were limited by a lack of training [64]. In France in 2017, a study indicated that out of 962 perinatal HPs, 74% recognize a lack of knowledge to address environmental health with their patients [65]. In fact, in a survey of 752 French GPs, only one out of four doctors reported having had training in environmental health (5% as part of their initial training and 21% as part of continuing education) [66]. 

We observed that HPs’ habits influence their MEDPREVED strategy adoption and prevention practice [67].

We observed that female HPs were more likely to respond to our survey.

We observed that the beginning of discussions was not encouraged by HPs faced with low-income patients. Some HPs did not integrate environmental health in their preventive practices because this topic was poorly comprehended. Indeed, HPs could consider that health subjects are the prerogative of affluent populations [37,68,69,70,71], a phenomenon which is described in the literature on stereotypes and discrimination [72].

We observed that the topic was not prioritized by HPs as they think scientific data are not robust enough and they prefer their prevention comfort zones, such as the elimination of tobacco consumption [73]. Lack of time and fear of provoking anxiety, notably from paediatricians who see pregnant women as vulnerable, are other dissuasive factors. The education of pregnant women about ED requires a positive discourse, as we have argued elsewhere [11].

### 3.8. Availability and Accommodation

Availability and accommodation refer to geographic location, accommodation, hours of opening, appointment mechanisms.

Geographical location positively influenced it accessibility, given the fact that equal shares of the population in the waiting rooms surveyed were living in rural and urban areas. A limitation factors could have been the medical density in our region. It may decrease further for a few years, making access to, and availability for, consultations more difficult. The share of prevention in general practices is currently estimated to exceed more than 30% of the general practitioner’s working time [74], which remains low and could be impacted by the decrease in general practitioners in some areas.

Accommodation (waiting room) is an essential point of this study whereas, in the pandemic period patient waiting time decreased [75], which may have impacted the MEDPREVED poster visibility and the time taken to complete the PREVED questionnaire.

MEDPREVED strategy allowed patients to take care of themselves before their consultation facilitating patient patience. We found that a lack of time and fees-for-service are not conducive to clinical prevention practices, specifically health education as described in the literature [76] and not all HPs grasp the waiting room as a prevention site [77], whereas it is an appropriate place to deliver prevention messages and promote health from both the physicians’ and patients’ perspectives [22,78,79]. Indeed, several studies have underlined the efficacy of this prevention tool: Besera et al. [64] showed in the USA that a low-resource video intervention for waiting rooms can provide sufficient exposure to positively influence sexually transmitted disease-related attitudes/behaviours; Eubele et al. [80] showed in Belgium that exposure to an audio-visual message about anti-tetanus vaccination was associated with an increase in the number of tetanus vaccine prescriptions. Several authors showed that video-formatted oral hygiene health education dental waiting rooms was effective in educating patients and instigating both immediate and sustained self-reported behaviour change (reduction in sweets consumption, reducing cavity rates) [52,81,82]. Single-session, video-based interventions can be highly cost-effective when implemented at scale [83].

If waiting rooms are salutogenic-designed, with plants reducing stress [84] or visual art [85,86], prevention information can increase patient awareness of certain health topics. Without salutogenic-design, posters alone are not necessarily effective in behaviour change [87,88]. However, as is shown in a French study on 60 HPs, none of them have a clearly defined strategy, mostly because they do not know how to act after risk factor screening [22,89]. In our study, HPs had the freedom to choose the document from the prevention kit that could be made accessible also in waiting rooms, as recommended by some authors [90].

### 3.9. Affordability

Affordability refers to direct, indirect and opportunity costs. MEDPREVED strategy is fee-free. It hypothesizes that as HPs do not have enough time during consultation, waiting time should be used with a self-administrated questionnaire. These tools (paper or online questionnaires, software) in primary care can be of help for the HP in overall patient management and follow-up, with early identification and a more rapid assessment of risk behaviours. Their utilisation has recently increased in Canada [91].

However, some respondents of our study proposed a hetero-administrated questionnaire containing the remarks and attitudes of the patients. Even if France belongs to the group of countries with the longest primary care consultation time, with an average of 16 min (compared to an average of 7 min for Germany and Spain) [92], there is clearly a lack of HP availability to address ED. In the French context of fee-for-prevention clinical practices, if adopted, the hetero-administrated questionnaire would be very quick. For example, HPs could ask two questions to each patient who, such as “Do you smoke?” and “Do you want to quit smoking?”; such questions have significantly increased the smoking cessation success rates [93,94]. This brief intervention in likewise effective with regard to physical exercise [95], fruit and vegetable consumption [96] or alcohol consumption in different populations [97,98,99]. HPs can use the 5A tool (Assess, Advise, Agree, Assist, Arrange) to support behaviour respecting the patient’s priorities and autonomy [100,101]. The structure of the PREVED questionnaire, both in its layout and in its length or simplicity of understanding, has made it accessible to many patients and could be utilized again in a brief intervention.

### 3.10. Appropriateness

Appropriateness refers to technical and interpersonal quality, adequacy, coordination, and continuity.

Whereas the interviewed HPs seemed to practice a global, biopsychosocial medicine, focusing on the patient-centred approach, excluding the traditional attitude of full control with a passive patient, encouraging the patient to be empowered [102], our main result was that the initiation of a discussion after the filling-in of the PREVED questionnaire was rare. The principal reason seems to be the lack of medical training in environmental health [103,104] and the wish to adapt an adaptive discourse [65,105,106,107,108,109] before using practical tools.

The MEDPREVED strategy is not inseparable from medical training. It could be improved by adding prevention kits to the QR code and delivering a paper with the QR code or internet site link at home for the whole family [110].

## 4. Strengths and Limits

The mixed method, with the guarantee of a collection of anonymised data, was appropriate, favouring the free expression of feelings, highlighting opinions on the device and objectifying practices. In qualitative studies, data saturation was obtained by the number of interviews carried out [111] and triangulation of the data during the analysis made it possible to limit interpretation errors. Interviews with participants were sometimes conducted at a distance from the completion of the questionnaire. As a result, loss of information was sometimes possible. In order to mitigate this limitation, some interviews were conducted just after the PREVED questionnaire was completed. There was also a selection and recruitment bias. Indeed, the participants may not be representative of the general population or of French health professionals. They may have been interested in the subject beforehand, whether they are professionals or patients.

## 5. Conclusions

The simplified MEDPREVED strategy is mostly feasible, accessible, and useful in clinical prevention practices. Compared to Levesque dimensions, at patient and HP levels, the MEDPREVED strategy has confirmed the same limiting factors and levers to access to ED prevention: need perception, ability to seek, reach and use ED exposure prevention. Analysis of this strategy could improve access to endocrine disruptor exposure prevention. The teachable moment could occur in every care setting. Further study is needed to measure the impact on knowledge, risk perception and behavior of beneficiaries of the MEDPREVED strategy in the medium and long term.

## Figures and Tables

**Table 1:** Characteristics of qualitative study population (M: midwife; Pe: Paediatrician or GP with a prenatal activity; P: patient from general population; Pa: patient with young children or pregnant woman).

Population	Age (Years)	Sex	Location	Characteristics	Number of Children
M1	53	Female	Urban	Private working for, 28 years	NA
M2	51	Female	Urban	Private working for 28 years	NA
M3	39	Female	Urban	Private working for 15 years	NA
M4	35	Female	Rural	Private working for 15 years	NA
M5	57	Female	Rural	Private working for 35 years	NA
M6	28	Female	Rural	Private working for 15 years	NA
M7	44	Female	Rural	Private working for 13 years	NA
M8	26	Female	Rural	Private working for 2 years	NA
M9	51	Female	Urban	Private working for 28 years	NA
M10	42	Female	Urban	Private working for 18 years	NA
M11	56	Female	Urban	Mother-and-child protection, working for 36 years	NA
M12	60	Female	Urban	Mother-and-child protection, working for 39 years	NA
Pe1	37	Female	Rural	Private GP, working for 8 years	1
Pe2	31	Male	Semi-rural	Hospital, working for 2 years	0
Pe3	55	Female	Semi-rural	Hospital, working for 25 years	2
Pe4	45	Female	Urban	Mother-and-child health, working for 7 years	?
Pe5	36	Male	Rural	Private GP, working for 8 years	2
Pe6	34	Male	Semi-rural	Hospital, working for 4 years	3
Pe7	50	Female	Rural	Private, working for 20 years	NA
Pe8	34	Female	Semi-rural	Hospital, working for 4 years	3
P1	43	Female	Semi-rural	Clinical psychologist	3
P2	21	Female	Rural	Child care worker	0
P3	36	Female	Rural	Start-up manager	1
P4	50	Female	Rural	Orderly	2
P5	49	Female	Rural	Dental assistant	3
P6	34	Female	Semi-rural	Life aid	2
P7	40	Male	Rural	Teacher	0
P8	20	Female	Rural	Student	0
P9	40	Female	Rural	Psychologist	3
P10	23	Female	Rural	Construction manager	0
P11	47	Male	Semi-rural	Mutual Trade Advisor	2
Pa1	26	Female	Rural	Rental Consultant	1
Pa2	33	Female	Rural	Farmer	1
Pa3	21	Female	Urban	Orderly	1
Pa4	33	Female	Urban	Associate professor at the university	0
Pa5	41	Female	Urban	Client Banking Advisor	1
Pa6	36	Female	Rural	Trainer in Rural Family Home	1
Pa7	34	Female	Rural	School nurse	0
Pa8	26	Female	Rural	Primary teacher/kindergarten	1
Pa9	30	Female	Semi-rural	Pulmonologist	1
Pa10 *	37	Female	Urban	Psychologist	1
Pa11 *	34	Male	Urban	Physics and chemistry teacher	1
Pa12	35	Female	Rural	hospital pharmacist	1

* couple; NA: not available; ?: M: midwife; Pe: Paediatrician or GP with a prenatal activity; P: patient from general population; Pa: patient with young children or pregnant woman).

**Table 2:** Characteristics of the 26 GPs included in the quantitative study.

	N	%
Sex		
Male	10	38
Female	15	58
Data missing	1	4
Department of the Nouvelle-Aquitaine region		
Charente (16)	5	19
Charente-Maritime (17)	3	12
Corrèze (19)	1	4
Dordogne (24)	2	7
Gironde (33)	8	31
Deux-Sèvres (79)	6	23
Haute-Vienne (87)	1	4
Medical practice location		
Rural	8	31
Semi-urban	11	42
Urban	7	27
Type of practice		
Medical office alone	4	15
Medical office group	15	58
Pluriprofessional medical center	7	27
Master of General Practice Internship	14	54
Has small children	13	50
Already knew about endocrine disruptors	19	73
Wanted training on the topic	26	100
Thought it was an important topic	25	96
Had already addressed the topic in consultation	15	58

**Table 3:** Feasibility, accessibility and utility of MEDPREVED strategy from General Practitioners’ point of view (n = 26).

	Not at All	Rather No	Rather Yes	Absolutely
Accessibility to PREVED© questionnaire by QR code is appropriate, n (%)	3 (18)	3 (18)	11 (65)	0
Numerical response modalities are easy, n (%)	0	3 (18)	14 (82)	0
Questionnaire is time-consuming, n (%)	0	4 (29)	9 (64)	1 (7)
It is feasible in routine with the same modalities (waiting room, QR code), n (%)	1 (7)	7 (50)	4 (29)	2 (14)
It is suitable for all patients, n (%)	0	5 (29)	12 (71)	0
The number of questions is adequate, n (%)	0	0	13 (77)	4 (24)
Questions are easy to read by your patients, n (%)	0	2 (12)	15 (88)	0
It has been appreciated by patients, n (%)	1 (8)	0	11 (85)	1 (8)
It introduced the topic of ED exposure, n (%)	1 (8)	1 (7)	7 (50)	5 (36)
It allowed you to improve your knowledge, n (%)	1 (8)	3 (21)	4 (29)	6 (43)
He encouraged you to engage in research, n (%)	0	2 (14)	9 (64)	3 (21)
It has changed the behaviour of some patients, n (%)	2 (14)	4 (29)	6 (43)	2 (14)

## Data Availability

All verbatims are available from Marion Albouy.
